# Apoptosis and Redistribution of the Ro Autoantigen in Balb/c Mouse Like in Subacute Cutaneous Lupus Erythematosus

**DOI:** 10.1080/17402520600876796

**Published:** 2006

**Authors:** Rafael Herrera-Esparza, Ricardo Villalobos, Juan-Jose Bollain-y-Goytia, Roxana Ramírez-Sandoval, Sergio H. Sánchez-Rodriguez, Guadalupe Pacheco-Tovar, Esperanza Avalos-Diaz

**Affiliations:** Department of Immunology Centro de Biología Experimental Universidad Autónoma de Zacatecas Zacatecas Mexico

## Abstract

In subacute cutaneous lupus eryhematosus (SCLE) the cutaneous antigens constitute the main source of Ro and La autoantigens. The aim of this investigation was to demonstrate if UV light increases the availability of Ro autoantigen in the skin, also the blocking effect of Ac-DEVD-CMK a caspase inhibitor was assessed. For this purpose newborn Balb/c mice were UVB irradiated (5–30 mJ/cm^2^) equivalent to a moderate to severe sunburn. Animals were injected with monoclonal anti-Ro antibodies from SCLE patients. Apoptosis was also induced by anti-Fas antibody injection. Skin samples were examined by direct immunofluoresence, by TUNEL, and the expression of caspase 3 by RT-PCR. Major findings of present studies were: 1. UVB irradiation and anti-Fas induced apoptosis of keratinocytes. 2. Apoptosis redistribute the Ro antigen on cell surface and is better triggered by Ro antibody. 3. The caspase 3 inhibitor Ac-DEVD-CMK decreases the availability of Ro autoantigen in epidermis and prevents deposition of anti-Ro. In conclusion, the caspase pathway would be blocked to avoid anti-Ro deposition along skin; this finding would be a prospect in the treatment of SCLE patients.


*Clinical & Developmental Immunology*, June–December 2006; 13(2–4): 163–166

Taylor & Francis

Taylor & Francis Group

### Apoptosis and redistribution of the Ro autoantigen in Balb/c mouse like in subacute cutaneous lupus erythematosus

RAFAEL HERRERA-ESPARZA, RICARDO VILLALOBOS, JUAN-JOSE BOLLAIN-Y-GOYTIA, ROXANA RAMÍREZ-SANDOVAL, SERGIO H SÁNCHEZ-RODRIGUEZ, GUADALUPE PACHECO-TOVAR, & ESPERANZA AVALOS-DIAZ

*Department of Immunology, Centro de Biología Experimental, Universidad Autónoma de Zacatecas, Zacatecas, Mexico*

**Abstract**
In subacute cutaneous lupus eryhematosus (SCLE) the cutaneous antigens constitute the main source of Ro and La autoantigens. The aim of this investigation was to demonstrate if UV light increases the availability of Ro autoantigen in the skin, also the blocking effect of Ac-DEVD-CMK a caspase inhibitor was assessed. For this purpose newborn Balb/c mice were UVB irradiated ($5-30\,mJ/cm^2$) equivalent to a moderate to severe sunburn. Animals were injected with monoclonal anti-Ro antibodies from SCLE patients. Apoptosis was also induced by anti-Fas antibody injection. Skin samples were examined by direct immunofluoresence, by TUNEL, and the expression of caspase 3 by RT-PCR. Major findings of present studies were: 1. UVB irradiation and anti-Fas induced apoptosis of keratinocytes. 2. Apoptosis redistribute the Ro antigen on cell surface and is better triggered by Ro antibody. 3. The caspase 3 inhibitor Ac-DEVD-CMK decreases the availability of Ro autoantigen in epidermis and prevents deposition of anti-Ro. In conclusion, the caspase pathway would be blocked to avoid anti-Ro deposition along skin; this finding would be a prospect in the treatment of SCLE patients.

**Keywords:** *Lupus, Ro antigen, apoptosis, cutaneous lupus*

### Introduction

Casciola-Rosen et al. demonstrated that autoantigens in lupus lacked of restriction to any subcellular location and demonstrated that under apoptosis, dying cells exhibited intracellular autoantigens on the cell surface throughout a physiologic process (Casciola-Rosen et al. 1994). In photosensitive subsets of lupus erythematosus has been recognized that apoptotic epidermal cells constitute a possible source of autoantigens; whereas in systemic lupus eythematosus (SLE) the apoptotic endothelial cells and lymphocytes probably contribute to trigger terminal-organ damage. Apoptosis guarantees the cellular exchange; therefore a healthy individual produce $1 \times 20^9$ Kg of cellular debris which is cleaned by phagocytes; an inappropriate cleaning of apoptotic material would triggers autoantibody production in individuals genetically predisposed (Lorenz et al. 2000). The epidermal release of intracellular antigens would induce *in situ* the formation of immune complexes that are related with the relapses of skin lesions, in consequence, the study of the factors that activate apoptosis are of clinical interest because would allow to propose therapeutic strategies to obtain clinical recovery. The aim of present study was to induce a redistribution of the Ro antigen of the skin in newborn Balb/c mice under UV irradiation, such redistribution was monitored by anti-Ro antibodies injected intraperitoneally. Additionally, we assess in the skin the effect of the caspase inhibitor Ac-DEVD-CMK on Ro/anti-Ro immune complex formation.

### Material and methods

### *Balb/c mice groups*

Animals were injected with anti-Ro antibody and controls with normal IgG. Mice were grouped in number of six according the following conditions: (A)

Correspondence: R. Herrera-Esparza, Department of Immunology, Centro de Biología Experimental, Universidad Autónoma de Zacatecas, Chepinque 306, Col. Lomas de la Soledad, Zacatecas 98040, México. E-mail: herrerar@intranet.uaz.edu.mx

ISSN 1740-2522 print/ISSN 1740-2530 online © 2006 Taylor & Francis
DOI: 10.1080/17402520600876796

Non-irradiated group. (B) UVB irradiated group. (C) UVB irradiated treated with Ac-DEVD-CMK. (D) Injected with anti-Fas to induce apoptosis. (E) Injected with anti-Fas and 2 h later injected with anti-Ro. (F) Anti-Fas treated with Ac-DEVD-CMK and then injected with anti-Ro. Animals were injected intra-peritoneal using a syringe in volumes adjusted to 50 μl. The antibodies were administrated in a dosage of 5 mg/g of body weight. The caspase inhibitor Ac-DEVD-CMK dissolved in DMSO adjusted to 20 mM was adjusted in PBS a final volume of 50 μl. A monoclonal anti-Ro antibody obtained from CLB (The Netherlands), or a polyclonal anti-Ro autoantibody from a SCLE patient, were used in all assays. The monoclonal anti-Fas antibody was obtained from Santa Cruz Biotechnology.

### *UV irradiation*

Was carried out by means of a UV lamp (Black-Ray UVL). The total dose of UV was 5–30 mJ/cm$^2$, which is equivalent in humans to a moderate to severe sunburn.

### *Skin biopsies*

Samples were obtained by means of a 2.5 mm punch, and were used for immunohistology and for RNA and DNA extraction by TRIsol, the epidermis was obtained by splitting with 10% dispase (Sigma). *Direct immunofluorescence* was done in a 4-μm slice of skin, and immune deposition was detected by FITC-rabbit anti human polyclonal IgG, or FITC-rabbit anti-Mouse (Sigma) in animals injected with monoclonal anti-Ro. *TdT-mediated dUTP nick end labelling* (*TUNEL*): Nuclear stripping was performed with 10 mM Tris–HCl, pH 8.0 followed by 15 min in 20 μg/ml proteinase K. Elongation of DNA fragments was done by incubation with reaction mixture (DDW, 10XTdT buffer (30 mM Tris base, 140 mM sodium cacodylate, pH 7.2, 1 mM cobalt chloride, 1 mM DTT; 10% of the final volume), fluorescein-11-dUTP (0.5 mg dissolved in 1 ml of 10 mM Tris–HCl, pH 7.0), and TdT enzyme (0.3 enzyme units/μl). Reaction was terminated by adding 300 mM NaCl, 30 mM sodium citrate, and pH 8.0. Finally the slides were evaluated under fluorescent microscopy to differentiate the true green tag of apoptotic cells from background incorporation, cells were counterstained with 0.5% propidium iodide, giving the non-apoptotic nuclei develop a red stain. *Oligonucleotides*: Caspase 3 forward 5′-TCC AGT CGG AGG CCA GAT CTG AG-3′, backward 5′-CTG AAG CCT GCC TCC CGG GAT GA-3′ [SNP000005036] and G3PDH forward 5′-TGA AGG TCG GTG TGA ACG GAT TTG GC-3′, backward 5′-CAT GTA GGC CAT GAG GTC CAC CAC-3′ (Clontech).

### *Reverse-transcription/polymerase chain reaction (RT-PCR)*

RNA (250 ng) was mixed with 200 μM dNTP and 0.7 μM of the backward primer and 5 U/20 μl of rTth/DNA polymerase and incubated at 70°C for 10 min. Amplification of caspase 3 and G3PDH cDNAs was carried out by PCR by addition of 0.15 μM of the forward primer; using 30 cycles of 94°C for 2 min, 48°C for 2 min and 72°C for 1.4 min. PCR products were electrophoresed in 0.8% agarose containing 0.5 mg/ml of ethidium bromide, and were observed under UV light. Caspase 3 was also detected by immunoperoxidase staining using an anti-caspase 3 antibody (Santa Cruz Biotechnology).

### Results

Non-irradiated animals did not show epidermal apoptosis; meanwhile UVB irradiated and anti-Fas injected animals exhibited apoptotic features induced in keratinocytes; such phenomenon was larger in UVB irradiated animals. The expression of caspase 3 by RT-PCR and immunohistochemistry of skin was evident in animals UVB irradiated or treated with anti-Fas antibody. On the other hand, the inhibitory effect of Ac-DEVD-CMK on apoptosis was remarkable, since apoptotic features were completely abolished in animals under treatment with this caspase 3 inhibitor. Under apoptosis Ro antigen became detectable in epidermis, therefore apoptosis redistribute the Ro antigen on cytoplasm and cell surface; therefore apoptotic keratinocytes were better triggered by the monoclonal or polyclonal anti-Ro antibodies. As expected the caspase 3 inhibitor Ac-DEVD-CMK decreased the availability of Ro autoantigen in epidermis, such decrease resulted in abolishment of anti-Ro deposition in epidermis, therefore skin biopsies of animals treated with this caspase 3 inhibitor resulted negative. Controls injected with normal human IgG were negative (Figure 1).

### Discussion

Main results of present study demonstrate that UVB irradiation and anti-Fas induces apoptosis of keratinocytes and Ro antigen translocation to cell surface, this facilitate the anti-Ro antibody triggering. We also show that Ac-DEVD-CMK decreases the availability Ro autoantigen and the anti-Ro deposition on the skin. In lupus sun exposure induces apoptosis and would contribute to the pathogenesis of skin lesions in patients with cutaneous lupus phenotype (Kelly et al. 2006; Kuhn et al. 2006). The release of intact or modified intracellular ribonucleoproteins including Ro60 (Ramirez-Sandoval et al. 2003) promotes the dendritic cell maturation and the production of interferon, that is critical in autoantibody production

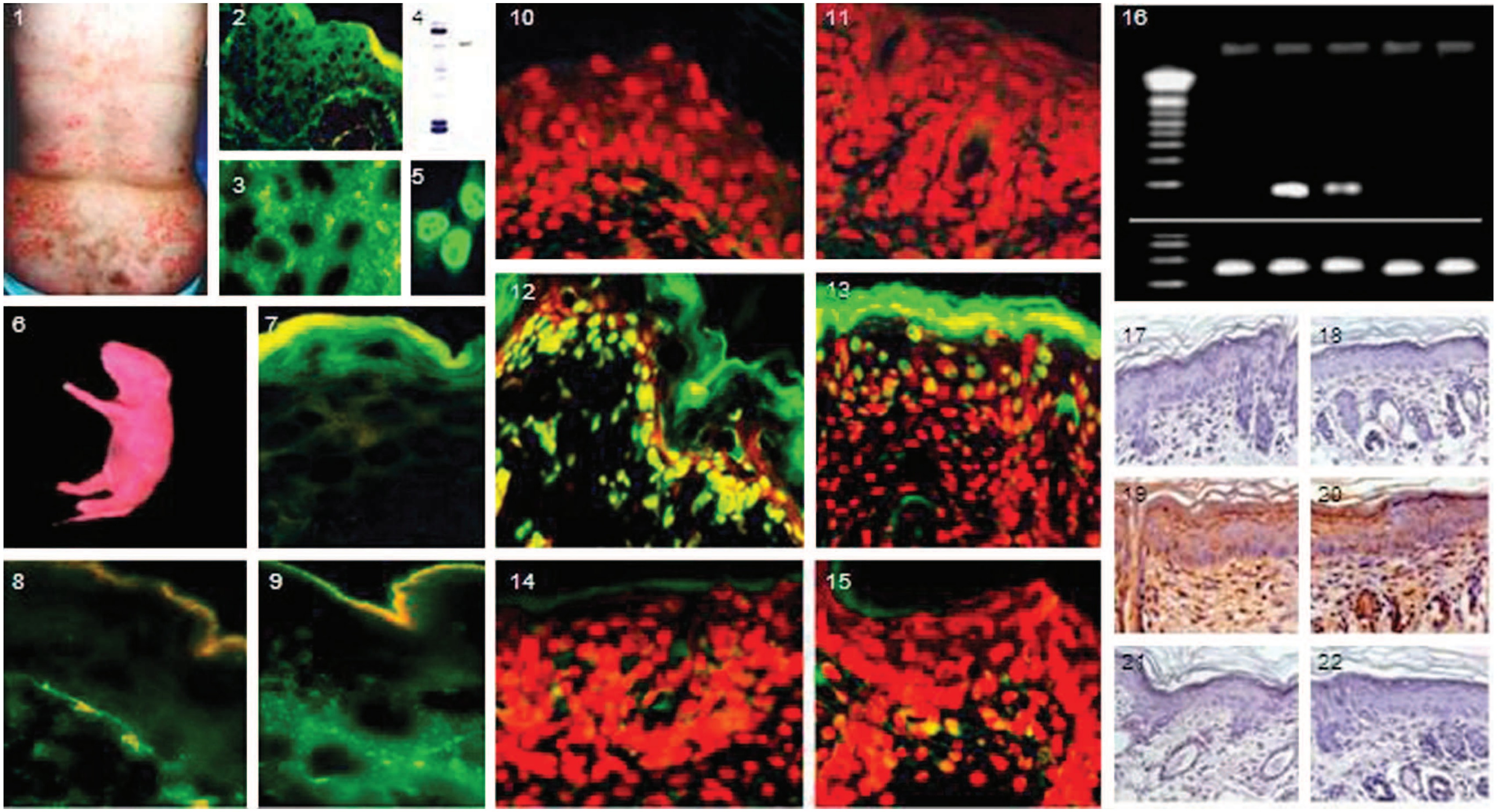

Figure 1. SCLE patient. 2. Skin biopsy of SCLE by immunofluorescence showing lupus band of IgG. 3. SCLE with IgG deposition in cytoplasm of keratinocytes. 4. Anti-Ro60 of the SCLE detected in human spleen extract by Western blot. 5. Positive ANA of the same patient. 6. Balb/c mouse injected with the human anti-Ro antibody from the SCLE patient. 7. Mouse skin biopsy non-UVB irradiated negative for IgG deposition. 8. UVB irradiated mouse show IgG resembling a lupus band. 9. UVB irradiated mouse show anti-Ro deposition in cytoplasm and cell surface of keratinocytes. 10–15 TUNEL assays: 10 and 11 controls. 12. UVB irradiated mouse show epidermal apoptotic green tag. 13. Anti-Fas induced apoptosis. 14. Absence of apoptosis in UVB irradiated mouse treated with caspase 3 inhibitor. 15. Absence of apoptosis in anti-Fas injected mouse treated with caspase 3 inhibitor. 16. RT-PCR amplification of caspase 3 (line$_1$ DNA markers, line$_2$ negative control, line$_3$ UVB irradiated mouse, line$_4$ anti-Fas injected mouse, line$_5$ UVB irradiated treated with caspase 3 inhibitor, line$_6$ anti-Fas injected and treated with caspase 3 inhibitor). In the inferior part of the gel the G3PDH housekeeping controls. 17–22 show the caspase 3 detection by immunoperoxidase. 17 and 18 negative controls. 19. UVB irradiated mouse shows extensive caspase 3 expression in epidermis and dermis. 20. Anti-Fas injected mouse with extensive expression of caspase 3. 21. UVB irradiated mouse treated with caspase 3 inhibitor. 22. Anti-Fas injected mouse treated with caspase 3 inhibitor.

and lupus relapses (Dall'era et al. 2005; Meller et al. 2005). Additionally, apoptosis induces secondary necrosis and chemokine production that is followed by recruitment and activation of autoimmune T cells and IFN-alpha-producing plasmacytoid dendritic cells creating a vicious circle. The local anti-Ro antibody production and effectors cytokines and leukocyte recruitment leads the development of cutaneous lupus lesions. The importance of present observation is that a caspase 3 inhibitor is capable to block the release of intracellular ribonucleoproteins such as Ro that is involved in SCLE pathogenesis; this experimental therapy has been successfully used to ameliorate kidney disease in a transgenic mouse model of lupus nephritis (Seery et al. 2001).

### Acknowledgements

Supported by CONACYT grant 38054-N and PROMRP 103.5/04/2310.

### References

Casciola-Rosen LA, Anhalt G, Rosen A. 1994. Autoantigens targeted in systemic lupus erythematosus are clustered in two populations of surface structures on apoptotic keratinocytes. J Exp Med 179:1317–1330.

Dall'era MC, Cardarelli PM, Preston BT, Witte A, Davis JC, Jr. 2005. Type I interferon correlates with serological and clinical manifestations of SLE. Ann Rheum Dis 64:1692–1697.

Kelly KM, Zhuang H, Nacionales DC, Scumpia PO, Lyons R, Akaogi J, Lee P, Williams B, Yamamoto M, Akira S, Satoh M, Reeves WH. 2006. "Endogenous adjuvant" activity of the RNA components of lupus autoantigens Sm/RNP and Ro 60. Arthritis Rheum 54:1557–1567.

Kuhn A, Herrmann M, Kleber S, Beckmann-Welle M, Fehsel K, Martin-Villalba A, Lehmann P, Ruzicka T, Krammer PH, Kolb-Bachofen V. 2006. Accumulation of apoptotic cells in the epidermis of patients with cutaneous lupus erythematosus after ultraviolet irradiation. Arthritis Rheum 54:939–950.

Lorenz HM, Herrmann M, Winkler T, Gaipl U, Kalden JR. 2000. Role of apoptosis in autoinmunity. Apoptosis 5:443–449.

Meller S, Winterberg F, Gilliet M, Muller A, Lauceviciute I, Rieker J, Neumann NJ, Kubitza R, Gombert M, Bunemann E, Wiesner U, Franken-Kunkel P, Kanzler H, Dieu-Nosjean MC, Amara A, Ruzicka T, Lehmann P, Zlotnik A, Homey B. 2005. Ultraviolet radiation-induced injury, chemokines, and leukocyte recruitment: An amplification cycle triggering cutaneous lupus erythematosus. Arthritis Rheum 52:1504–1516.

Ramirez-Sandoval R, Sanchez-Rodriguez SH, Herrera-van Oostdam D, Avalos-Diaz E, Herrera-Esparza R. 2003. Antinuclear antibodies recognize cellular autoantigens driven by apoptosis. Joint Bone Spine 70:187–194.

Seery JP, Cattell V, Watt FM. 2001. Cutting edge: Amelioration of kidney disease in a transgenic mouse model of lupus nephritis by administration of the caspase inhibitor carbobenzoxy-valyl-alanyl-aspartyl-(beta-*o*-methyl)-fluoromethylketone. J Immunol 167:2452–2455.

